# Optimization of Antioxidant and Skin-Whitening Compounds Extraction Condition from *Tenebrio molitor* Larvae (Mealworm)

**DOI:** 10.3390/molecules23092340

**Published:** 2018-09-13

**Authors:** Julie J. Kim, Kyoung Seob Kim, Byung Jo Yu

**Affiliations:** 1 Department of Overseas Study Program, Hanyoung Foreign Language High School, Dongnam-ro 832, Kangdong-gu, Seoul 05279, Korea; juliehs0522@gmail.com; 2 Division of Material Development, ByTheLab. Cosmetics, 7-2, 710 ho, KwongKyo Central Rd., Yongtong-gu, Suwon-si 16512, Korea; greensky-s@daum.net; 3 IT Convergence Materials R&BD Group, Korean Institute of Industrial Technology, 89 Yangdaegiro-gil, Ipjang-myeon, Seobuk-gu, Cheonan-si 31056, Chungnam, Korea

**Keywords:** *Tenebrio molitor*, phenolic compound, antioxidant, whitening, extraction, optimization

## Abstract

Skin-whitening ingredients are a very important part of the development of functional cosmetics and a wide variety of raw materials are used. Tyrosinase is a key enzyme in the animal melanogenic pathway that is the rate-limiting step for the production of melanin. Several synthetic and naturally occurring tyrosinase inhibitors have been studied for skin-whitening. The development of natural agents is becoming more important due to the disadvantages of synthetics such as high cytotoxicity, insufficient penetration power, and low activity. The purpose of this study was to evaluate the total phenol content (TPC), antioxidant, and tyrosinase inhibition activity of mealworm (*Tenebrio molitor* larvae) extract, and the subsequent optimization of the extraction condition using statistically-based optimization. The major extraction variables extraction temperature, time, and ethanol concentration were optimized using response surface methodology (RSM). The results showed that optimum extraction temperature of 88.1 °C, extraction time of 43.7 min, and ethanol concentration of 72.0 *v*/*v*%, provided the predicted maximum levels of total phenolic compounds (TPC) of 5.41 mg GAE/g dry weight (DW) and tyrosinase inhibition activity (TIA) of 82.4%. From the validation experiment, 5.61 ± 0.2 mg GAE/g dry weight (DW), tyrosinase inhibition of 79.6 ± 3.3%, and radical scavenging activity of 91.8 ± 5.1 μg/mL were found and showed to be very similar to the predicted values. These results suggest that mealworm has great potential as a source of bioactive compounds which could be used as cosmetics, food, and pharmaceutical agents.

## 1. Introduction

The world’s rapid population growth resulted in a global food shortage, especially of animal protein sources [1]. Other factors such as competition between humans and livestock over crops and environmental problems are raising concerns, so insects are gaining more attention as an alternative protein and an alternative source of value-added materials [2,3]. Insects have a lower proportion of cholesterol and are a good source of vital minerals, vitamins, lipids, and carbohydrates [4,5]. Amino acid composition, (such as leucine, lysine, and isoleucine) within mealworms meet both the requirements of animals and human beings. Despite the advantages of consuming alternative proteins, eating insects is still considered socially uncomfortable and such issues remain to be solved. Therefore, there are more benefits for using edible insects for cosmetic materials in skin care products than for food.

During several decades, the cosmetic and medical industries have been focusing their research on treating skin disorders. A number of studies have proved that the extracts and isolated compounds from numerous natural sources and synthetics have great potency in skin-whitening and anti-aging activity. Tyrosinase is known as a copper-containing enzyme involved in the formation of melanin pigments in mammals; therefore, controlling tyrosinase is important for the purpose of whitening effects [6]. Recently, tyrosinase inhibitors have become important, as they are used as depigmentation ingredients of medical and cosmetic products [7]. Tyrosinase is mainly involved in two distinct reactions of melanin synthesis; first, the hydroxylation of tyrosine to 3,4-dihydroxyphenylallanine (DOPA) and second, the conversion of DOPA to the corresponding dopaquinone. Dopaquinone undergoes several reactions to eventually forms melanin [8].

Even though numerous synthetic tyrosinase inhibitors have been reported, only a few of them such as arbutin and kojic acid are commercially used, mainly due to disadvantages like high cytotoxicity, insufficient penetrating power, low activity, and low stability [9,10]. Hence, there is a great demand for the development of natural skin-whitening agents, which are free from side-effects. The extracts and isolated compounds of numerous natural sources, in particular botanical sources, were well-characterized with anti-tyrosinase activities and have been accepted as skin-whitening agents [11,12,13]. Reviews of potent tyrosinase inhibitors were mainly focused on various plants rather than animals. Table 1 shows the tyrosinase inhibitory activity of natural extracts of plants and also animals.

Many reviews of potent tyrosinase inhibitors derived from various plants and animals have motivated us to study the tyrosinase inhibition of insects, especially edible insects, which are widely produced and safe in terms of food security. Among the few insect studies, there have been reports of the inhibition of tyrosinase by silkworm extracts [20]. However, mealworm, a rising source of food commercially reared in many countries, has also been reported to contain antioxidant activity [21,22,23].

The scope of this study was a thorough investigation on the potential of green solvents including water and ethanol for the extraction of skin-whitening agents and phenolic compounds from mealworm based on RSM. Therefore, a three-variable five-level central composite design (CCD) was used to optimize and study the effects of extraction temperature, extraction time, and ethanol concentration on the maximum TIA and TPC from mealworm.

## 2. Results

### 2.1. Opimization of Extraction Variables

A three-variable five-level CCD consisting of 17 experimental runs was used with three center points. In order to reduce the effects of unexplained variability, the experiment was conducted in random order. The independent variables were extraction temperature (X_1_, °C), extraction time (X_2_, min), and ethanol concentration (X_3_, *v*/*v*%). The TPC and TIA of all 17 runs are shown in Table 2. Among these 17 experiments, experiments 8 and 14 showed the highest TPC and TIA levels, respectively, while experiments 15 and 9 produced the least TPC and TIA levels, respectively. This result indicates that the extraction tendency of TPC and TIA is uncoupled and different optimum conditions are expected to exist. Since it was found that the quadratic model for the experimental data better than other models, an ANOVA was used to determine the best correlation between independent variables and dependent variables (TPC and TIA) of the quadratic models. The regression coefficients with *p* < 0.05 are highly significant, whereas the terms having insignificant response coefficients with a *p* > 0.1 are least contributing in the prediction of *Y*_1_ and *Y*_2_. The insignificant terms were removed from the full model to generate the reduced model equation. The quadratic equation was obtained by omitting the term $x_{2}x_{3}$ with *p* > 0.1. Therefore, a reduced model was chosen as follows:

(1) $$Y_{1}=5.00+0.671x_{1}+0.195x_{2}+0.431x_{3}+0.396x_{1}x_{2}+0.327x_{1}x_{3}-0.651x_{1}{}^{2}-0.375x_{2}{}^{2}-0.796x_{3}{}^{2}$$

(2) $$Y_{2}=79.55+6.74x_{1}+10.37x_{2}+9.83x_{3}-4.70x_{1}x_{2}-3.16x_{2}x_{3}-3.71x_{1}{}^{2}-14.68x_{2}{}^{2}-13.79x_{3}{}^{2}$$

where the coefficients $x_{1}$, $x_{2}$, and $x_{3}$ correspond to hot-water extraction variables of extraction temperature, time, and ethanol concentration in coded values. *Y*_1_ = TPC; *Y*_2_ = TIA.

The coefficients of each first-order regression equation provide a measure of the effect level of the independent variable on the response. The quadratic models (Equations (1) and (2)) showed that the linear terms of $x_{1}$, $x_{2}$, and $x_{3}$ were all positive and the three variables showed proportional effects on the responses (TPC and TIA). Therefore, this means that as the three dependent variables increase, the two independent variables also increase. An ANOVA of independent variables listed in Table 3 shows that the main variables, including extraction temperature (X_1_), extraction time (X_2_), and ethanol concentration (X_3_), were all significant (*p* < 0.05) and exhibited positive effects on TPC and TIA in the following orders of X_1_ > X_3_ > X_2_ and X_2_ = X_3_ > X_1_. The model’s goodness-of-fit was checked by determination coefficient (R^2^). The value of R^2^ closer to 1 denoted better correlation between the observed and predicted responses. An R^2^ value less than 80% indicates that the model does not sufficiently explain the relationship between the experimental variables [24]. In our models, the high R^2^ values, 0.9719 and 0.9638, for TPC and TIA, respectively, imply the experimental data confirm the predictability with the data predicted by the model.

**Figure 1:**
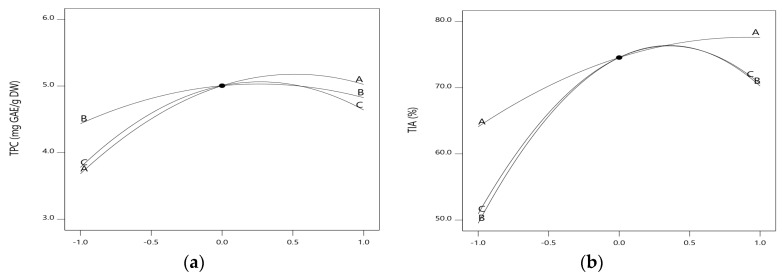
(**a**) Perturbation plot of actual TPC of mealworm extracts as a function of extraction temperature, extraction time, and ethanol concentration; (**b**) Perturbation plot of actual TIA of mealworm extracts as a function of extraction temperature, extraction time, and ethanol concentration.

As shown in Figure 1, perturbation plots described the comparative effects of all the experimental variables at the midpoint at a coded value of 0.0 in the design space. The curvatures with all three variables, temperature, time, and ethanol concentration show that all these variables exert different degrees of effect on the response. However, the curve with the most dramatic change was the perturbation curve of ethanol concentration compared to those of the other two. Thus, it is evident that concentration of ethanol significantly contributed to the extraction of TPC and TIA and showed the most pronounced quadratic effect.

The effective way to evaluate the effect of the independent variables on the responses is to plot response surface curves, which was done by varying two variables within the experimental range while fixing one variable at the central point. To investigate the interactive effects of variables for the TPC and TIA values, three-dimensional plots were generated based on Equations (1) and (2) while keeping the concentration of ethanol fixed at 61.3 *v*/*v*%. The R^2^ values for the TPC and TIA models are very good, and the ANOVA table suggests that the models fitted well by first order (FO), two-way interaction (TWI), and second order (SO) terms. For both models the FO, TWI, and SO terms appear to be significant, but only the X_2_X_3_ term appears to be insignificant. Since the FO and TWI terms appear to be significant, the graph showed a linearity and a curved property for TPC and TIA.

**Figure 2:**
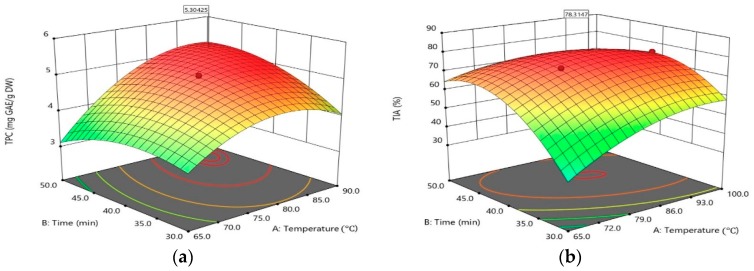
(**a**) Surface response curve on extraction of TPC of mealworm extract. Interactive effects of extraction temperature and extraction time at fixed level of ethanol concentration of 61.3 *v*/*v*%; (**b**) Surface response curve on TIA of mealworm extract. Interactive effects of extraction temperature and extraction time at fixed level of ethanol concentration of 61.3 *v*/*v*%.

As shown in Figure 2a, the three dimensional response surface plot obtained from the RSM study shows the combined effect of extraction temperature and time on the actual TPC when ethanol concentration was kept constant at 61.3 *v*/*v*%. The maximum TPC could be obtained with an extraction temperature of 86.3 °C and extraction time of 46.4 min. The saddle point was found within the range of experiments, as two-way interaction variables were contributing to optimizing the extraction. The temperature increase in the extraction would destroy the extracted ingredient and it would be better to keep the temperature at 86.3 °C for the future TPC extraction experiments. Figure 2b shows the effect of extraction temperature and time on the TIA of the mealworm extract. The level of TIA increased with increasing extraction time and temperature. Then, it tends to decrease again after passing the maximum point. The maximum TIA of 78.3% could be predicted with extraction temperature of 86.3 °C and 42.4 min at a concentration of ethanol of 61.3 *v*/*v*%. According to Figure 2a,b, the increase in temperature improves the extraction of TPC because of the enhanced movement of molecules during solvent extraction at high temperature, as a result of higher diffusion coefficients and greater solubility of compounds. However, above a certain level of temperature and time, TPA and TIA tend to decline again, which is expected to be caused by a decomposition of bioactive materials at high temperatures [25]. Also, under a long extraction period, it could be because TPC and TIA of the mealworm extract have reached an equilibrium concentration during solvent extraction and further extracting could provide a chance for the exposure to heat, oxygen, and UV which enhances the degradation process of bioactive materials [26].

**Figure 3:**
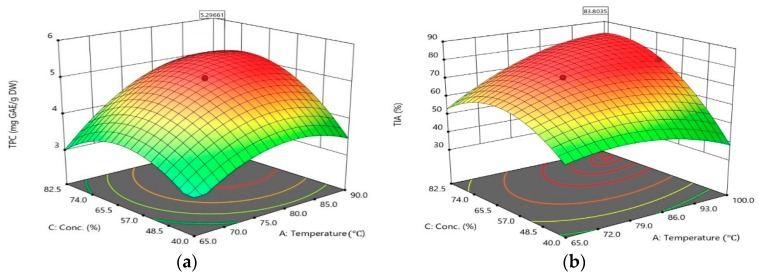
(**a**) Surface response curve on TPC of mealworm extract. Interactive effects of extraction temperature and ethanol concentration at fixed level of extraction time of 40 min; (**b**) Surface response curve on TIA of mealworm extract. Interactive effects of extraction temperature and ethanol concentration at fixed level of extraction time of 40 min.

The yield of TPC and TIA, as a function of extraction temperature and ethanol concentration, was graphically represented in three-dimensional surface plots as showed in Figure 3a,b. According to ANOVA, all tested FO variables have significant effects on TPC and TIA. For the extraction of mealworm, ethanol concentration (*p* = 0.0004, *p* = 0.0003) and extraction temperature (*p* = 0.0001, *p* = 0.0031) have significant impacts on the extraction of TPC and TIA, respectively. It is observed that the yield of TPC gradually increases with increasing temperature at a high concentration of ethanol, while the curve for ethanol concentration concaves downwards, indicating that the yield of TPC decreases beyond the optimum concentration of a certain ethanol level at all levels of temperature (Figure 3a). When temperature and ethanol concentration increased, the level of TIA firstly increased and then began to decrease at the arrival of the maximum point. The optimal conditions for the highest TIA was 97.9 °C and 77.8 *v*/*v*% ethanol at fixed level of extraction with 40 min duration of reaction, under which TIA was the highest of 83.8%. Thus, the extraction yield of ethanol/water mixture is higher than the solvent using 97 *v*/*v*% ethanol. It is thought that the polarity of the mixture of water and ethanol was suitable for extracting bioactive material from mealworm. According to the results from Waszkowiak and Gliszczyńska-Świgło [27], the extraction method with 60 *v*/*v*% ethanol can be an efficient and simple method to produce flaxseed extract that could be utilized as a valuable food ingredient such as phenolic compounds. These data were consistent with the conclusion of the direct effect of these variables in Figure 1. The order of relative importance of these three variables was found as temperature ≥ ethanol concentation > time and time ≥ ethanol concentation > time.

### 2.2. Process Optimization and Validation

**Figure 4:**
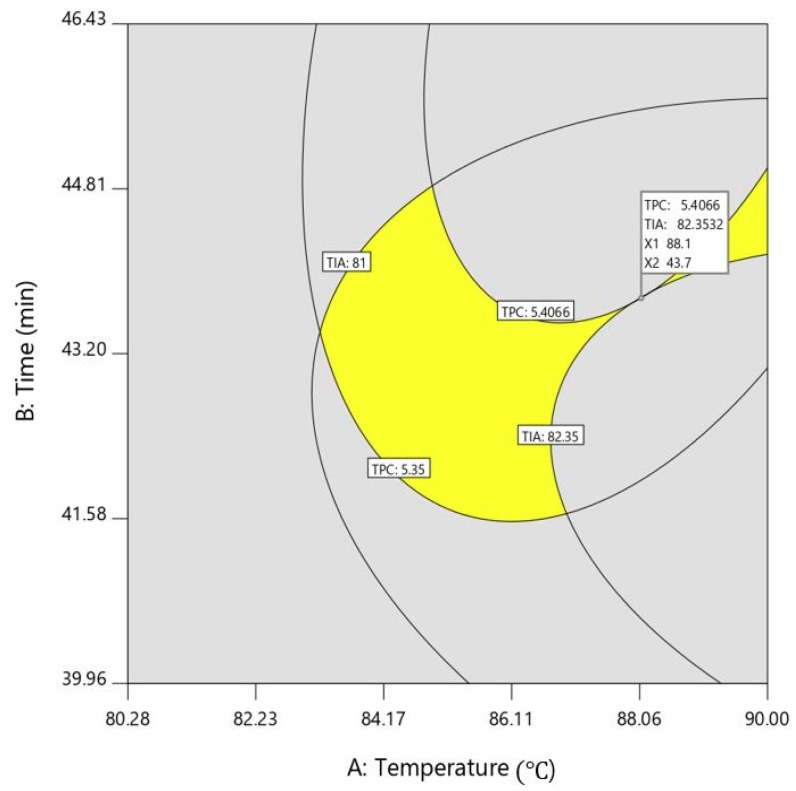
Overlay plot for the optimal region (best combination) of the extraction temperature and time, for the prediction of maximum levels of TPC and TIA.

Because the optimal conditions of two responses are different, a superimposed contour plot was employed to optimize the extraction conditions for extraction temperature, time, and ethanol concentration to simultaneously obtain maximum concentrations of TPC and TIA. In our previous study, in order to apply a superimposed contour plot, one independent variable was set at a fixed level to evaluate the effect of the other two variables on dependent responses. Thus, a superimposed counter plot (Figure 4) for TPC and TIA as a function of temperature and extraction time was plotted, with the ethanol concentration fixed at the optimum level of 72.0 *v*/*v*%. The results showed that TPC of 5.41 mg GAE/g DW and TIA of 82.4% were predicted as optimum conditions under the extraction variables of extraction temperature of 88.1 °C, extraction time of 43.7 min. To verify the quality of the result obtained from RSM, the validation experiment was conducted in triplicate and compared with levels of TPC and TIA predicted under optimum conditions. The levels of TPC and TIA of the validation experiment were 5.6 ± 0.2 GAE/g dry weight (DW) and 79.6 ± 3.3, respectively. When the *t*-test was performed to assess the statistical significance of predicted and validation values, the difference between the two values was not significant with *p* = 0.143 and *p* = 0.5 for TPC and TIA, respectively, implying that no significant difference was found between validation and predicted values. It was shown that optimized hot-water extraction by statistical optimization was superior to Sohxlet extraction under the same conditions. Thus, the RSM used for the optimization of extraction conditions of TPC and TIA is a very useful tool for predicting the response values.

### 2.3. Antioxidant Activity (Radical Scavenging Activity)

In order to confirm the antioxidant effects of mealworm extract, the proton radical scavenging activities of mealworm extracts were measured by DPPH assay which is widely used to evaluate antioxidant activity in a relatively shorter time of analysis than other methods. In the previous 17 experiments for the production of TPC (Table 2), three extracts (#15, #8, and #14) and the extract from the optimized condition were chosen for DPPH assay. Table 4 shows the IC_50_ whose radical scavenging capacity was found to exhibit a 50% of inhibition value. The scavenging effect of mealworm extracts on DPPH radicals increases along with TPC concentration. For the DPPH radical, the extraction with optimized conditions showed the lowest radical scavenging activity of 91.8 ± 5.1 μg/mL based IC_50_. This result showed that the higher antioxidant activity (lower IC_50_) of the mealworm is in correlation with its higher TPC. In our experiment, the antioxidant effect of mealworm extract was proved and it is expected that the higher level of radical scavenging activity of mealworm would be obtained by the extraction process optimization. It was also concluded that water/ethanol extraction of mealworm possesses antioxidant and tyrosinase inhibition activities and thus it can be applicable as a potent natural antioxidant in cosmetics, food, and pharmaceutical industries for the preparation of value-added products.

## 3. Discussion

Mealworm contains bioactive compounds including phenolic compounds, peptides, and fatty acids that in many plant extracts were proved to have evident cosmetic, pharmaceutical, and medical care function such as antioxidant and tyrosinase inhibitions activities. In this study, phenolic compounds in mealworm were extracted by hot-water extraction using binary solvent and extraction conditions optimized using the RSM. The optimization results showed that the second-order polynomial model gave a satisfactory prediction of the TPC and TIA results. Optimal extraction conditions were found to occur when the following set of variables were used. Temperature = 88.1 °C, extraction time = 43.7 min, and ethanol concentration of 72.0 *v*/*v*%. Under the conditions, TPC, TIA, and antioxidant potential have the highest levels of 5.6 ± 0.2 mg GAE/g DW. TIA of 79.6 ± 3.3 *v*/*v*% (IC_50_ = 4.4 ± 0.3 μg/mL) and DPPH of 91.8 ± 5.1 μg/mL in the validation experiment, respectively. In conclusion, our results showed that water–ethanol binary solvent extract of mealworm has phenolic compounds, tyrosinase inhibition activity, and proton radical scavenging potentials which are comparable to other natural extracts, thus it could be used for antioxidant and skin-whitening materials. These results suggest that mealworm extract is novel and useful as a natural source of bioactive compounds for cosmetics, food, and pharmaceutical materials. In this experiment, only a total phenolic compounds measurement was performed, and in future studies, it would be necessary to identify each phenolic compound which has bioactive properties. Also, additional studies are necessary to develop a method for the fractionation and identification of mealworm phenolic compounds and to increase the antioxidant activity in mealworm extracts.

## 4. Materials and Methods 

### 4.1. Mealworms

Freeze-dried yellow mealworm (*Tenebrio molitor*) larvae were purchased from the local mealworm farm (Wellbeing Mealworm Farm, Asansi, Korea). In the farm, the worms were raised on sawdust with organic feed which mainly consists of fish and wheat powder. The freeze-dried larvae were ground to a powder using a food processor (Hanil, HMF-3800, Seoul, Korea) with four blades. Because particle size is a fundamental parameter for solid-liquid extraction, ground larvae were sifted through a 60-mesh screen. Then, the powder was stored in a sealed zipper bag at −20 °C before extraction and analysis.

### 4.2. Extraction

Hot-water extraction using binary solvent of water–ethanol was used to extract the organic soluble compounds from mealworm. Extraction experiments were carried out in batch mode in a water bath with air-tight screw-cap borosilicate glass tubes (20 mL, Corning Inc., Corning, NY, USA). First, 0.5 g of dried mealworm and 10 ml of binary solvents (water–ethanol) were added to obtain a 1:20 solid to liquid ratio and the tubes were then placed in a water bath thermostated at the temperature values reported in Table 2 controlled at ±0.1 °C (Korea Science Co., CMS 393, Seoul, Korea). To prevent the evaporation of ethanol by heating during hot-water extraction, an air-tight high-pressure tube was used to prevent evaporation of ethanol. After completing the extraction, total weight of tube was measured and the tubes were selected only within 2 *v*/*v*% of ethanol evaporation and used for further analysis after calibration by adding distilled water. As shown in Table 5, one class of statistically-based optimization, RSM, was used to evaluate the effect of extraction temperature (X_1_; 56.5–98.5 °C), extraction time (X_2_; 23.2–56.8 min), and ethanol concentration (X_3_; 25.5–97.0 *v*/*v*%). At each sampling time, a sample was harvested, centrifuged, and separated by 0.45 μm disk filter (26 mm, Supelco, St. Louis, MO, USA) from remaining solid. Filtrate stored at −20 °C for TIA and TPC assay tests. Depending on the concentration of extracts, the filtered extracts were diluted 10–100 times prior to the assay.

### 4.3. Determination of Total Phenolic Compounds (TPC)

The concentration of TPC was determined using Folin–Ciocalteu method with gallic acid as a standard [23]. Briefly, an aliquot (100 μL) of each diluted extract was added to 0.25 mL of the Folin–Ciocalteu reagent, the mixture was vortexed and allowed to reaction in a static condition. After 5 min, 1 mL of 2 *w/v*% sodium carbonate solution was added. After 60 min of incubation in dark at room temperature, the absorbance was measured at 650 nm. The TPC was expressed as mg of gallic acid equivalent per gram of dry weight mealworm (mg GAE/g DW).

### 4.4. Determination of Tyrosinase Inhibition Activity (TIA)

The effect of mealworm extracts on TIA was measured spectrophotometrically with DOPA as a substrate [28]. The reaction mixture (1000 μL) contained 685 μL of phosphate buffer (0.05 M, pH 6.5), 15 μL of mushroom tyrosinase (2500 μ/mL), 200 μL of mealworm extract solution, and 100 μL of 5 mM DOPA. The reaction was measured at 480 nm for the production of the dopachrome product after the addition of DOPA. Kojic acid was used as a positive control and the inhibition of tyrosinase activity was calculated with the following equation:

(3) $$\mathrm{Inhibition}\;\left(\%\right)=\frac{1-\left(A_{480}\;in\;sample\right)}{A_{480}\;in\;control}\times 100\%$$

### 4.5. DPPH Radical Scavenging Assay

The ability of mealworm extract to scavenge the 1,1-diphenyl-2-picrylhydrazyl (DPPH) radical was measured by the procedure of Saha and Verma [29]. The addition of 0.1 mM DPPH solution in various concentrations (10, 25, 50, 75, 100, and 150 μg/mL) of mealworm extract with Tris–HCl buffer (50 mM, pH 7.4) and the absorbance was measured at 520 nm. A mixture of methanol and extract used as the blank. The IC_50_ values for the mealworm extract were calculated and compared with the standard reference compound ascorbic acid.

### 4.6. Experimental Design

A CCD of a three-variable five-level was used to optimize extraction variables such as extraction temperature, extraction time, and ethanol concentration. Experimental design, data analysis and model building have been conducted using Design Expert software and ANOVA was applied to analyze the results. Each factor was coded at five-level according to the following equation,

(4) $$\mathrm{xi}=\frac{\left(X_{i}-Xz\right)}{\Delta X_{i}}$$

where xi is the dimensionless coded value of an independent variable; *X_i_* is the actual value of an independent variable; *Xz* is the actual value of an independent variable at the center point; and Δ*X_i_* is the step change of the actual value of the variable (*i* = 1, 2, 3, 4, 5).

The range and center point values of the independent variables presented in Table 5 The experimental data obtained from CCD was analyzed by using RSM to fit the following second-order polynomial equation (Equation (5)).

(5) $$Y=\beta_{0}+\overset{k}{\underset{i=1}{\sum }}\beta_{i}X_{i}+\overset{k}{\underset{i=1}{\sum }}\beta_{ii}X_{i}{}^{2}+\overset{k-1}{\underset{i=1}{\sum }}\overset{k}{\underset{j=2}{\sum }}\beta_{ij}X_{i}X_{j}$$

where *X_i_* is the encoded independent variable affecting the response *Y*; *β*_0_, *β_i_* (*i* = 1, 2,…, *k*), *β_ii_* (*i* = 1, 2,…, *k*) and *β_ij_* (*j* = 1, 2,…, *k*) are the regression coefficients for intercept, linear, quadratic, and interaction terms, respectively; *k* = 3; *i* < *j*.

The Design-Expert version 8.0 (Stat-ease Inc., Minneapolis, MN, USA) software was used for the development of regression and prediction of responses. The quality of the model’s fitness and significance was evaluated using the coefficients of determination (R^2^) and analysis of variance (ANOVA). Response surfaces curves were developed using the fitted second-order regression model.

## Figures and Tables

**Table 1:** Tyrosinase inhibitory activity of natural extracts [14,15,16,17,18,19,20].

	IC_50_ Value (mg/mL)	Extract Solvent
Camellia pollen	0.05	ethanol
Thai mango seed kernel	98,630	ethanol
*Blumea balsamifera DC* leave	0.319, 0.345	Hexane, water
*Euphorbia lathyris* L.	0.28	methanol
Peel of Citrus Fruit	2.42	methanol
Far Eastern sea cucumber	0.49–0.61, 1.80–1.99	Ethanol, water
Sericin extract of silkworm	1200–18,760	water

**Table 2:** Experimental runs of the central composite design and their responses, including actual and predicted values of total phenol compounds (TPC) and tyrosinase inhibition activity (TIA).

	X_1_	X_2_	X_3_	Actual		Predicted	
				*Y*_1_	*Y*_2_	*Y*_1_	*Y*_2_
Run#	Temp. (°C)	Time (min)	Ethanol Conc. (*v*/*v*%)	TPC (mg GAE/g DW)	TIA (%)	TPC (mg GAE/g DW)	TIA (%)
1	77.5	40.0	61.3	5.07	75.2	5.01	74.6
2	65.0	50.0	40.0	2.37	46.2	2.21	47.8
3	77.5	40.0	61.3	4.92	73.3	5.01	74.6
4	65.0	30.0	40.0	2.86	19.7	2.61	17.7
5	77.5	40.0	61.3	5.04	74.7	5.01	74.6
6	65.0	50.0	82.5	2.74	49.6	2.41	53.6
7	77.5	40.0	97.0	3.54	56.9	3.48	52.1
8	90.0	50.0	82.5	5.09	61.2	5.20	71.6
9	77.5	40.0	25.5	1.87	17.1	2.03	19.0
10	65.0	30.0	82.5	2.73	22.1	2.82	23.4
11	77.5	23.2	61.3	3.76	13.3	3.62	15.7
12	90.0	30.0	40.0	2.33	22.2	2.50	26.7
13	90.0	50.0	40.0	3.76	43.6	3.69	38.0
14	98.5	40.0	61.3	4.55	76.3	4.30	75.5
15	56.5	40.0	61.3	1.68	54.7	2.04	52.7
16	77.5	56.8	61.3	4.04	55.7	4.28	50.6
17	90.0	30.0	82.5	3.84	66.2	4.02	60.2

**Table 3:** Analysis of variance (ANOVA) of the TPC and TIA.

	TPC				TIA	
	Sum of Squares	*F* Value	*p* Level	Sum of Squares	*F* Value	*p* Level
Model	20.64	27.54	0.0001	7687.94	29.10	<0.0001
X_1_	6.15	73.86	<0.0001	620.95	21.15	0.0025
X_2_	0.5211	6.26	0.0409	1467.43	49.98	0.0002
X_3_	2.53	30.43	0.0009	1320.85	44.99	0.0003
X_1_X_2_	1.26	15.08	0.0060	177.02	6.03	0.0438
X_1_X_3_	0.8563	10.29	0.0149	387.31	13.19	0.0084
X_2_X_3_	0.0133	0.1602	0.7009 *	79.91	2.72	0.1430 *
X_1_^2^	4.79	57.48	0.0001	155.31	5.29	0.0550
X_2_^2^	1.59	19.05	0.0033	2428.74	82.73	<0.0001
X_3_^2^	7.14	85.77	<0.0001	2145.15	73.07	<0.0001

*p* value less than 0.05 indicates model term is significant. In this case X_1_, X_2_, X_3_, X_1_X_2_, X_1_X_3_, X_1_², X_2_², X_3_² were significant model terms. Only two-way interaction * X_2_X_3_ was insignificant model term; total phenol compounds (TPC); tyrosinase inhibition activity (TIA).

**Table 4:** Radical scavenging activity of mealworm extracts extracted by ethanol solution.

	Temp. (°C)	Time (min)	Conc. (*v*/*v*%)	TPC (mg GAE/g DW)	TIA (%)	DPPH (IC_50_, μg/mL)
Run #15	56.5	40.0	61.3	1.68	54.7	394.3
Run #8	90.0	50.0	82.5	5.09	61.2	161.8
Run #14	98.5	40.0	61.3	4.55	76.3	188.5
Optimized condition	88.1	43.7	72.0	5.41	82.4	-
Validation experiment	88.1	43.7	72.0	5.6 ± 0.2	79.6 ± 3.3 (4.4 ± 0.3 μg/mL) *	91.8 ± 5.1
Soxhlet extraction	88.1	43.7	72.0	2.3 ± 0.2	53.6 ± 2.2	190.6 ± 8.2
Ascorbic acid	-	-	-	-	88.9	40.2 ± 1.8

Concentrations of extracts and ascorbic acid were adjusted at 250 μg/mL for scavenging activity tests. DPPH was for radical scavenging activity test. The values of the validation experiment were obtained from validation experiment under the optimized conditions. * IC_50_ values (μg/mL) by tyrosinase inhibition assay.

**Table 5:** The factors and levels of central composite design.

	−1.68	1.0	0	1.0	1.68
Extraction Temperature (°C)	56.5	65.0	77.5	90.0	98.5
Extraction Time (min)	23.2	30.0	40.0	50.0	56.8
Ethanol Concentration (*v*/*v*%)	25.5	40.0	61.3	82.5	97.0
